# Peripheral S100B Protein Levels in Five Major Psychiatric Disorders: A Systematic Review

**DOI:** 10.3390/brainsci13091334

**Published:** 2023-09-16

**Authors:** Tomasz Kozlowski, Weronika Bargiel, Maksymilian Grabarczyk, Maria Skibinska

**Affiliations:** 1 Student’s Research Group “Biology of the Neuron”, Department of Psychiatric Genetics, Poznan University of Medical Sciences, 60-806 Poznan, Poland; 2 Protein Biomarkers Unit, Department of Psychiatric Genetics, Poznan University of Medical Sciences, 60-806 Poznan, Poland

**Keywords:** S100B protein, schizophrenia, major depressive disorder, bipolar disorder, depression, autistic spectrum disorder, attention-deficit/hyperactivity disorder

## Abstract

Five major psychiatric disorders: schizophrenia, major depressive disorder, bipolar disorder, autistic spectrum disorder, and attention-deficit/hyperactivity disorder, show a shared genetic background and probably share common pathobiological mechanisms. S100B is a calcium-binding protein widely studied in psychiatric disorders as a potential biomarker. Our systematic review aimed to compare studies on peripheral S100B levels in five major psychiatric disorders with shared genetic backgrounds to reveal whether S100B alterations are disease-specific. EMBASE, Web of Science, and PubMed databases were searched for relevant studies published until the end of July 2023. This study was performed according to the Preferred Reporting Items for Systematic Reviews and Meta-analysis Protocols (PRISMA) guidelines. Overall, 1215 publications were identified, of which 111 full-text articles were included in the systematic review. Study designs are very heterogeneous, performed mostly on small groups of participants at different stages of the disease (first-episode or chronic, drug-free or medicated, in the exacerbation of symptoms or in remission), and various clinical variables are analyzed. Published results are inconsistent; most reported elevated S100B levels across disorders included in the review. Alterations in S100B peripheral levels do not seem to be disease-specific.

## 1. Introduction

Shared genetic susceptibility in psychiatric disorders is widely studied in Genome-Wide Association Studies (GWAS). The degree to which genetic variation is unique to individual diseases or shared across conditions is still unclear. Comparative GWAS analyses consistently confirm correlations in five major psychiatric disorders: high between schizophrenia (SCH) and bipolar disorder (BD); moderate between SCH or BD and major depressive disorder (MDD), as well as MDD and attention-deficit/hyperactivity disorder (ADHD) and low between SCH and autistic spectrum disorder (ASD) [1,2,3]. The most recent study combining GWAS data with neuroimaging quantified the extent of shared genetic components between cortical structures and five major psychiatric disorders. Positive correlations in ADHD, BD, MDD, and SCH were found, while ASD was significantly correlated with ADHD, BD, and SCH [4]. Multi-trait analysis of GWAS studies by Wu et al. (2020) also confirmed shared genetic correlations between five major psychiatric disorders [5]. We can assume that shared genetic susceptibility underlies common biological mechanisms underlying pathological conditions in genetically related diseases. Specific polymorphisms have been associated with a range of psychiatric disorders. In particular, calcium-channel activity genes seem to have pleiotropic effects on psychopathology [1].

S100B is a calcium-binding protein belonging to the S100 protein family. The central nervous system (CNS) is expressed mainly in glial cells, especially astrocytes, but also in oligodendrocytes, as well as in Schwann, ependymal, and retinal Muller cells. S100Bb is not a glial-specific marker; its expression was also found in neuron subpopulations. Outside the CNS, S100B is expressed in enteric glial cells, adipose tissue, and lymphocytes [6]. S100B acts in a dose-dependent manner, with the opposite biological effect. Nanomolar doses have a neurotrophic action, while micromolar concentrations show pro-inflammatory and apoptotic effects [6]. S100B is a marker of blood–brain barrier integrity (BBB) [7]; however, elevated circulating S100B concentrations in neuropsychiatric disorders might be a consequence of altered peripheral expression rather than BBB disruption [8]. 

Numerous studies on circulating S100B concentration in various psychiatric disorders were performed up to date, with conflicting results. Our systematic review aims to compare results on circulating S100B levels in five major psychiatric disorders with shared genetic susceptibility: schizophrenia, bipolar disorder, major depressive disorder, attention-deficit/hyperactivity disorder, and autistic spectrum disorder. We present cross-sectional and longitudinal studies and analyze S100B levels regarding different clinical variables and pharmacological and non-pharmacological treatments.

## 2. Materials and Methods

This study was performed using a predetermined protocol in accordance with the Preferred Reporting Items for Systematic Reviews and Meta-Analysis (PRISMA) statement [9].

### 2.1. Search Strategy

EMBASE, PubMed, and Web of Science databases were searched for relevant studies published until the end of July 2023. The search terms were: [(S100B plasma) OR (S100B serum) OR (S100B circulating)] AND [(Schizophrenia) OR (Bipolar Disorder) OR (Major Depressive Disorder) OR (Depression) OR (attention-deficit/hyperactivity disorder) OR (Autism) OR (Autistic Spectrum Disorder)]. Duplicate records were removed automatically using EndNote X8.2 software, Clarivate, Philadelphia, PA. Manual screening of the remaining articles using title and abstract allowed the exclusion of non-inherent studies. Full-text screening of the remaining records was performed.

### 2.2. Inclusion Criteria 

Inclusion criteria: (a) full-text articles, (b) published in English, (c) published in peer-reviewed journals, (d) human studies, (e) cross-sectional, or longitudinal studies, or correlations of S100B with clinical parameters in clinical samples, and (f) meta-analyses

### 2.3. Exclusion Criteria

The exclusion criteria were as follows: (a) non-human studies, (b) expression of S100B in the brain (both at mRNA and protein level), (c) peripheral expression on mRNA level, (d) other neuropsychiatric diagnoses, (e) review articles, (f) retracted articles, (g) non-English articles, and (h) duplicates.

## 3. Results

### 3.1. Search Results

**Figure 1:**
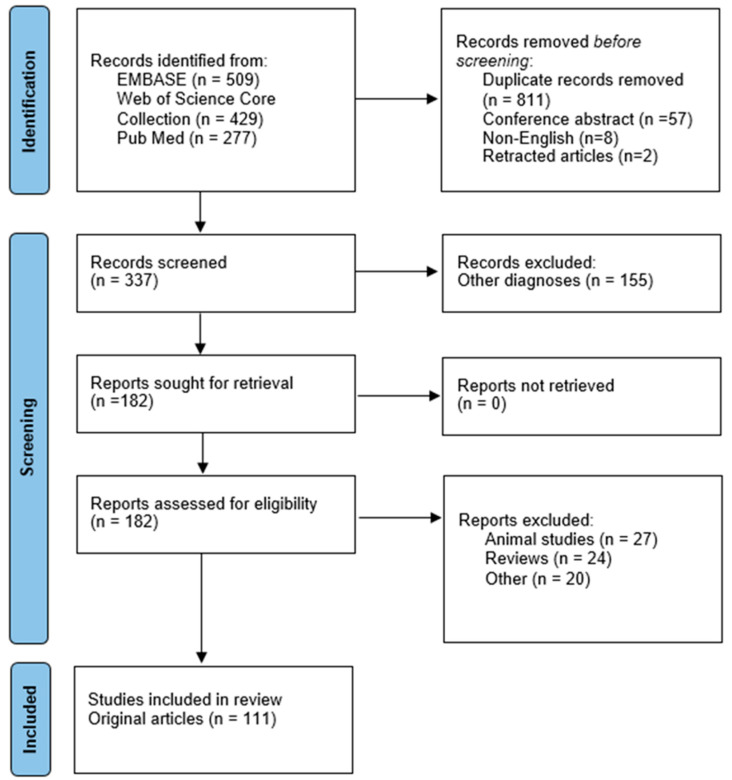
PRISMA Flow Diagram for the systematic review process.

A total of 1215 publications were identified, of which 182 full texts were evaluated (Figure 1). In the present systematic review, 111 studies were included.

### 3.2. Schizophrenia (SCH)

#### 3.2.1. Cross-Sectional Comparisons

Comparing circulating S100B levels between schizophrenia patients and matched healthy controls (HC), most studies reported elevated S100B, either in chronic or first-episode SCH. In some studies, both first-episode and chronic patients were involved. Elevated circulating S100B levels were detected in first-episode drug-naïve or drug-free SCH patients compared with HC [10,11,12,13,14,15,16,17,18,19,20,21,22,23,24,25]. Also, most studies revealed higher S100B in medicated patients than HC [10,23,24,26,27,28,29,30,31,32,33,34,35]. One report found elevated S100B only in female SCH compared to HC [36].

Results are not unequivocal because no differences between SCH and HC were reported [34,37,38]; also, lower S100B was detected in SCH compared to HC [39,40].

Opposite results concern comparisons between unmedicated (drug-naïve or drug-free) and schizophrenic patients medicated with neuroleptics (typical or atypical). Higher S100B levels were reported in drug-naïve (*n* = 80) compared to medicated (*n* = 82) SCH patients [15]. The contradictory effect was also found to be higher for S100B in medicated patients compared to unmedicated SCH patients [34,38].

#### 3.2.2. Longitudinal Comparisons

Changes in S100B levels during antipsychotic treatment were widely studied, with contrary results. No differences [13,23,39,41,42,43,44,45,46] or decreases [12,19,45,47,48,49] in S100B concentrations were reported during antipsychotic treatment. Elevation in S100B levels in the patient group with predominant negative symptoms were also found [39]. A similar result was reported by Sarandol et al. (2007) in a group of patients with negative symptoms after six weeks of treatment with various antipsychotic drugs where S100B levels were significantly reduced [13].

No differences in S100B between groups medicated with risperidone or flupenthixol were observed, though higher S100B levels were found in the group with poor improvement after 12 weeks of treatment [41]. Another study by Rothermundt et al. (2001) showed no differences in S100B levels between the patients treated for six weeks with typical or atypical neuroleptic drugs. No correlation between S100B and the dose of haloperidol equivalents was found [14].

Wu et al. (2018) followed a large group of schizophrenic patients (*n* = 93 in acute relapse, *n* = 97 in stable condition) for nine weeks of medication with different antipsychotics either in monotherapy or two or more antipsychotics prescribed. A significant decrease in S100B levels during treatment was detected [49]. The effect of adjuvant therapy was also investigated. In the double-blind controlled clinical trial with Withania somnifera (ashwagandha, WSA) vs. placebo as adjuvant therapy with antipsychotics, a more significant decrease in S100B was observed, along with a better improvement in negative symptoms, in the WSA group compared to placebo [47].

No differences in S100B concentrations in the groups of patients treated with typical or atypical neuroleptics were reported [15,32,33,41]. No correlation of S100B concentration with serum levels of antipsychotics drugs was found [32]. No differences were found in S100B levels between patients treated with clozapine and those with depot antipsychotic medication [36]. No correlation with the dose of antipsychotic drugs was found in all studies that reported such parameters [40,42,50,51].

#### 3.2.3. Correlation with Demographic and Clinical Parameters

Most studies reported the absence of correlation of S100B levels with age [10,13,15,30,33,35,36,40,50], but positive [32], as well as negative [43], correlations with age were discovered, also. There were no correlations of circulating S100B with gender in most of the studies published [10,13,15,22,27,30,33,40,50,52], although higher S100B levels were found in schizophrenic males compared to females [32], contrary to higher S100B in female SCH compared to male SCH [36].

No correlation of S100B levels with schizophrenia symptoms severity measured using Positive and Negative Symptoms Scale (PANSS) total score was detected in the majority of the studies [20,27,28,30,31,39,40,41,43,45,47,48,50,51]. In contrast, others reported a positive [17,19,21,23] or negative [22] correlation with total PANSS score. Additionally, positive correlations with the PANSS positive [10,12,17] and PANSS negative [12,23,41,42] subscales were found.

Wu et al. (2018) divided a large group of SCH patients (*n* = 190) into high and low S100B levels subgroups. In the high S100B group, higher baseline PANSS total, positive, negative, and general subscales scores were observed [49]. Van De Kerkhof et al. (2014), dividing the patient sample (*n* = 58) according to baseline low, middle, and high S100B levels, found that the subgroups with low and high S100B levels had higher PANSS total scores than the middle subgroup. In the whole patient sample, no correlation with symptom severity measured with PANSS was detected [39]. Higher S100B levels in patients with prevailing negative symptoms than in patients with positive symptoms were observed. Scale for Assessment of Negative Symptoms (SANS) and Scale for Assessment of Positive Symptoms (SAPS) were applied in the study [13].

A negative correlation with deficit symptoms [32] and negative symptoms measured with SANS [32] was reported, while a positive relationship with deficit symptoms was also detected [38].

No significant correlation between the levels of S100B and acute illness severity measured using the Brief Psychiatric Rating Scale (BPRS) [18,36] or negative symptoms severity (SANS) was found [36]. In the study by Schroeter et al. (2003), S100B was positively correlated with the subscore ‘thought disturbance’ of the BPRS [38].

Baseline S100B correlations with total and negative PANSS scores after 12 weeks of antipsychotic treatment were no longer present [23]. Higher PANSS negative scores at baseline and after treatment in patients with unchanged S100B levels during treatment were found [14].

Dai et al. (2020) found no differences in S100B levels in outpatients divided regarding dominant positive or dominant negative symptoms measured with PANSS [16]

Inconsistent results of peripheral S100B correlations with illness duration were published, including positive [16,22,37], absence [10,13,30,33,35], or negative [20] correlation with length of disease.

Zhang et al. (2010) applied the Abnormal Involuntary Movement Scale in dyskinetic patients and found a positive correlation with s100b serum levels [31].

Chronic SCH treatment-resistant patients (*n* = 63) treated with clozapine or atypical antipsychotics were involved in the study by Qi et al. (2009). There were no correlations between s100b levels and subtypes of schizophrenia, age at onset, and duration of taking neuroleptics [33].

In the study by Pinjari et al. (2022) on 106 schizophrenic inpatients, S100B positively correlated with soluble P-selectin, which was shown to play an essential role in the initial recruitment of leukocytes to the sites of injury during inflammation [53].

#### 3.2.4. Child/Adolescent Schizophrenia

Studies on groups of children and adolescents with early onset schizophrenia deserve special attention because there are fewer of them than studies on groups of adult patients. On the other hand, the early onset group has a more substantial neurodevelopmental component in the etiology of the disease; thus, from a biological point of view, it is more homogenous and easier to find relationships between biological factors and disease mechanisms. Elevated S100B serum levels in 28 children and adolescents with first-episode psychosis compared to eight healthy controls were reported, with no further analyses with clinical factors [25]. A study conducted by Zakowicz et al. (2023) on schizophrenia-spectrum adolescent inpatients (*n* = 45) and healthy controls (*n* = 34) found no differences in S100B levels either in exacerbations of psychotic symptoms or after 6–8 weeks of treatment. A negative correlation between age and the number of suicidal attempts was detected. A lack of correlation with symptom severity measured with PANSS, either at baseline or after treatment, was found [43]. Another study compared S100B levels between 60 children SCH patients treated with risperidone for 12 weeks, with 60 matched healthy controls. S100B serum levels were checked at baseline and after neuroleptic treatment. Baseline S100B levels were higher in SCH group, while after treatment, they were significantly lower compared to HC. Positive correlation between PANSS total score and s100b serum levels was detected [19].

#### 3.2.5. Cognitive Functions

First-episode (*n* = 40) and chronic (*n* = 35) schizophrenia patients were assessed for psychopathology and cognitive functions (Auditory Verbal Learning Test, AVLT; and Diagnostic Test of Cerebral Dysfunction). Each group was divided into high and low S100B subgroups. S100B levels did not change during treatment. The proportion of participants in the high S100B group was more prominent in chronic patients. A decrease in S100B during treatment was detected in both low S100B groups (first-episode, FEP and chronic SCH). Chronic SCH with high S100B had poorer verbal memory performance than chronic and FEP patients with low S100B levels. There were no differences regarding figural memory between the studied groups [45].

The cognitive functions of drug-free patients with schizophrenia (*n* = 78, onset of disease ≤ 3 years) were assessed by MATRICS Consensus Cognitive Battery (MCCB) and compared with 71 healthy controls. The speed of information processing, word learning, reasoning and problem-solving, and visual learning T-score of the SCH was lower than HC. The negative correlation of S100B with the speed of processing and verbal learning was detected in the patient group [17].

Chen et al. (2017) compared drug-naïve (*n* = 34), drug-free (*n* = 28) schizophrenia patients and HC with regard to S100B serum levels and MCCB results. The drug-free group had a longer disease duration, while in the drug-naïve group, higher values were detected in the PANSS total, positive and general subscores. In the drug-free group, serum S100B levels negatively correlated with the MCCB composite score, working memory, reasoning/problem-solving, visual learning, attention/vigilance, and processing speed and verbal learning. No significant associations between S100B and MCCB composite score or any cognitive domain subscore were observed in the drug-naïve group [10].

Chukaew et al. 41 chronic SCH patients in their study, all treated with antipsychotics for at least 12 months. The participants’ memory and learning functions, processing speed and attention, executive function, and intelligence were assessed. S100B serum levels negatively correlated with processing speed and attention [54].

Cognitive functions were assessed by the NIH Toolbox Cognition Battery in chronic, medicated schizophrenic patients (*n* = 39). The cognitive domains showed no correlations between S100B levels and the Flanker Inhibitory Control and Attention Test, Dimensional Change Card Sort Test, Picture Vocabulary, Fluid Composite, List Sorting Working Memory test, Pattern Comparison, Picture Sequence Memory test, and Oral Symbol Digit Test [28].

#### 3.2.6. Neuroimaging Studies

Milleit et al. (2016) performed a study combining voxel-based morphometry of white matter structures and S100B serum levels in 17 drug-free/naïve schizophrenic patients (first episode and recurrent episode) and 22 controls. Clusters indicating significant differences in the association between S100B concentration and white matter were found, such as a posterior cingulate bundle and temporal white matter structures assigned to the superior longitudinal fasciculus. S100B-associated alterations of white matter already exist at the first psychotic episodes, and are distinct from those in recurrent episode patients [11].

In the study by Van Der Leeuw et al. (2017), 32 patients with psychotic disorder, 44 non-psychotic siblings, and 26 HC were assessed by magnetic resonance imaging for cortical thickness (CT) and fractional anisotropy (FA). No correlations between serum S100B and brain measures were found [52].

#### 3.2.7. Seasonal and Day/Night Changes in S100B Levels in SCH

Serum S100B levels were determined at 12:00 and 24:00 h in SCH (*n* = 23) and HC (*n* = 23). Patients had significantly higher serum S100B levels measured at 12:00 h and lower levels of S100B measured at 24:00 h compared to HC, both at admission and discharge. Three months after discharge, patients and healthy subjects had similar S100B levels [55]. In a larger studied group, Morera–Fumero et al. (2017) found significantly higher S100B serum levels measured both at noon and midnight in schizophrenic inpatients (*n* = 65) during an acute episode compared to HC. Patients had higher S100B at 12:00 compared to 24:00, while in controls, S100B levels did not differ between day and night [56]. Another study showed higher S100B levels in SCH patients admitted to the hospital in winter compared to the summer group; the autumn group had intermediate S100B concentrations [57].

### 3.3. Major Depressive Disorder (MDD)

#### 3.3.1. Cross-Sectional Comparisons

Inconsistent results of cross-sectional comparisons of S100B levels in major depressive disorder (MDD) were reported. In first-episode MDD patients, an increase in circulating S100B levels compared to HC was found [58,59], while Yang et al. (2008) did not detect differences in first-episode MDD compared to HC. Similar results were obtained by Arora et al. (2017) on a group of adolescent and young adult patients [60,61].

There are only two studies exclusively on adolescent groups concerning S100B levels, and opposite results were reported: decreased [62] or elevated S100B levels [63] in adolescent MDD patients compared to HC.

Conflicting results are reported in MDD patients with longer illness duration. An increase in S100B levels [60,61,64,65], no differences [66,67,68,69,70,71], or lower [61,72,73] S100B levels in recurrent episodes of MDD compared to HC were found. Rothermundt et al. (2001) described a S100B increase in melancholic depression compared to HC and no differences in non-melancholic depression [74]. Fang et al. (2016) compared first-episode drug-naïve group as well as citalopram-treated patients with HC and found higher S100B in both patients’ groups compared to HC. No significant differences between drug-naïve and medicated patients were detected [59]. A lack of differences [59,73], or lower S100B in first-episode depression patients compared to recurrent depressive episode [60,61] was reported. Two studies of S100B levels in pregnant women treated with SSRI (*n* = 75) [75], or exclusively with paroxetine (*n* = 50) [76] found elevated S100B levels in both groups compared to HC.

#### 3.3.2. Longitudinal Comparisons

SRRI having no treatment effect on S100B was reported in a few studies [58,62,64,65,77].

Arolt et al.’s pilot study (2003) found a positive correlation of S100B at baseline in the melancholic patients’ subgroup with relative response to antidepressant therapy after four weeks [73]. Jang et al. (2008) found higher baseline S100B levels in responder (*n* = 30) compared to non-responder (*n* = 29) patients. The serum S100B level increased after six weeks of treatment, which was more prominent in non-responders than in responders. After treatment, the serum S100B levels of responders and non-responders baseline differences ceased [67].

Patients with moderate to severe depression (*n* = 40), after a week of wash-out, were randomized to treat with venlafaxine or imipramine. Measures were performed at baseline after seven weeks and six months of medication. Baseline serum S100B correlated with treatment response, and patients with high baseline S100B showed a more significant improvement in reducing depressive symptoms [78].

In the study by Navines et al. (2022), patients with high baseline S100B levels significantly improved Montgomery Åsberg Depression Rating Scale scores compared to those with low S100B levels during pharmacotherapy with escitalopram or sertraline [79]. No correlation of S100B with ketamine treatment of drug-resistant depression was found [80,81], nor correlation with suicidal ideation in ketamine-treated patients [81].

#### 3.3.3. Correlation with Demographic and Clinical Parameters

A lack of correlation of S100B levels with age in MDD patients was consistently reported [60,64,65,69,73,74].

Yang et al. (2008) found that female patients had higher S100B levels than male patients; no differences with regard to gender were observed in HC. Also, no differences between female MDD and HC, as well as male MDD and HC, were observed [60]. In adolescent groups, higher S100B levels were found in female patients compared to HC, and no differences were found between male and female MDD patients [61]. Some studies reported a lack of differences in S100B levels concerning gender [73,74].

Most of the studies reported a lack of correlation of S100B levels with symptoms severity using different clinical scales: Hamilton Depression Rating Scale (HDRS) [59,60,62,65,72,73,74,77,78], Clinical Global Impression (CGI), Mini-Mental State Examination (MMSE) [72]. Quick Inventory of Depressive Symptomatology Self-Report (QIDS-SR) [82], Beck Depression Inventory (BDI) [61], Hamilton Anxiety Scale (HAMA) scores [59], and Montgomery Åsberg Depression Rating Scale (MADRS) [79].

A positive correlation of S100B concentrations with HDRS scores in the inpatients with depressive episodes [64,68], and lack of correlation after treatment [64], was also reported. A negative correlation of S100B levels with baseline BDI and MADRS scores in treatment-resistant, severely depressed patients included in the ECT therapy were found [83].

Conflicting results are also obtained concerning age at onset, illness duration, and the number of episodes, and S100B levels. However, only a few studies included these variables in the analyses. No correlation with age at onset was reported by Yang et al. (2008) [60]. No relationships of S100B with illness duration nor with a number of episodes were found [65,73,74]. Conversely, a positive correlation with the number of depressive episodes, but not illness duration, was detected [60].

In the study by Jha et al. (2019), relationships between plasma S100B, anhedonia, and treatment response was studied in the group of *n* = 153 depressed patients treated with escitalopram in monotherapy, escitalopram + bupriopion, or venlafaxine + mirtazapine. Higher baseline S100B levels were correlated with smaller reductions in anhedonia in patients treated with escitalopram in monotherapy; no associations between baseline S100B levels and depression severity were found [82].

Wallensten et al. (2022) found that plasma levels of S100B were under ELISA detection limit in all HC (*n* = 61); only four female patients with MDD had detectable S100B levels; thus, results in such a small group are preliminary. Cognitive Failures Questionnaire scores and severity of depression measured with MADRS were positively correlated with levels of S100B. Plasma levels of astrocyte-derived extracellular vesicles were associated with levels of S100B [66].

Higher plasma S100B levels of the melancholic compared to the non-melancholic subsample of patients were reported [73,74], though both studies were performed at the same university. Thus, study groups are probably overlapping.

Bilginer et al. (2021), in a group of adolescent patients with MDD (*n* = 49), reported a negative correlation between the S100B level and the severity of anxiety measured with the Screen for Child Anxiety Related Disorders (SCARED). No correlations with severity of depression (Beck Depression Inventory; BDI) nor suicidal behavior (Suicide Probability Scale; SPS) scores were found [63]. Lack of association with stressful life events (measured with Brief Life Events Questionnaire; BLEQ) was found in adult MDD females [77].

Another study by Pawluski et al. (2019) on pregnant women showed that lower S100B levels at delivery were associated with higher maternal depression symptoms in SSRI-treated women. No relationship between S100B and maternal mood symptoms in non-SSRI-treated women was detected [84].

Some studies compared S100B levels between different diagnostic groups. Similar S100B levels were found in MDD and panic disorder patients [58]. No differences in serum S100B levels between patients with major depressive disorder, bipolar disorder, schizophrenia, and generalized anxiety disorder were detected, as well as in comparison with the control group [71].

A positive correlation of S100B concentration with family history of depression was discovered in adult [60] and adolescent [62] patients.

#### 3.3.4. Electroconvulsive Therapy (ECT)

Electroconvulsive therapy (ECT) is a non-pharmacological method of treating drug-resistant depression. Only some studies in this field have been conducted, and there is mixed evidence of its relation to S100B. Patients with intermediate S100B levels were more likely to achieve a remission of symptoms after ECT than patients with lower S100B levels. There also was no difference in S100B between baseline and post-ECT in both: remitters and non-remitters [85]. Palmio et al. (2010) found that a reduction in depressive symptoms after ECT treatment correlated with high S100B levels at 2 and 6 h post-ECT [86]. In the other study, it was demonstrated that remitters had higher baseline levels of S100B compared to non-remitters, but there were no significant changes in S100B from baseline to after-ECT measures in the whole group [83]. Gbyl et al. (2022) showed no correlation between baseline or post-ECT S100B change and clinical outcome; and no change in S100B levels both: shortly and six months post-treatment [87]. Elevation in S100B concentration one hour after ECT was observed in the study by Arts et al. (2006). Higher baseline S100B was correlated with poorer memory function at 30 days follow-up, but also with less subjective cognitive impairment and better response to treatment [88]. In another study, no changes in S100B during ECT were detected. S100B levels were not correlated with symptoms improvement or with alterations in cognitive performance. Baseline S100B serum levels were not associated with age, sex, BMI, diagnosis (MDD or BD), duration of illness or length of current episode, and comorbid dementia. S100B was not associated with the severity of depressive episodes or cognitive decline [89].

#### 3.3.5. Event-Related Potentials

Dietrich et al. (2004) followed currently remitted MDD patients (*n* = 12) for 12 weeks, with every 2 weeks of S100B examination. The matched healthy control group was involved in the study. No significant intra-individual changes in S100B levels were noticed during observation. Patients and controls were divided into “high” and “normal” S100B level groups. Event-related potentials (ERPs) during the Go/Nogo paradigm were investigated in relation to S100B. Patients with “high” S100B serum levels showed a normal N2- and P3 amplitude, while patients with “normal” S100B exhibited a reduced N2- and P3 amplitude of ERPs [69]. Another study on this group was performed concerning word memory processing. A continuous word recognition task was applied, and ERPs were recorded. Patients with moderately increased serum S100B levels showed a normal old/new effect. A reduced old/new effect was detected in patients with normal S100B levels [90].

ERPs and serum S100B levels were studied in patients with MDD before and after four weeks of treatment with citalopram or reboxetine. In patients with elevated baseline S100B levels, increased P3-latency normalized, and P2-latency significantly decreased after treatment [64].

#### 3.3.6. Other Interventions

In the study by Dai et al. (2018), elderly patients (*n* = 136) were randomly divided into two groups, and treatment with escitalopram or escitalopram + gingko biloba extract was administered for 12 weeks. A positive correlation with the severity of depression, persistent error number in the Wisconsin Cards Sorting Test, and baseline S100B serum levels were detected. A decrease in S100B levels in both groups after treatment was noticed, with a more significant effect for combined therapy (ginkgo biloba extract + escitalopram) [91].

Serum S100B was analyzed in 22 patients with depression who received repetitive transcranial magnetic stimulation (rTMS) for three weeks with ultra-high frequency stimulation (*n* = 14) or sham (*n* = 8). No effect of treatment on S100B was detected [92].

Blood-to-brain and blood-to-CSF perfusion rates in depressed patients and healthy controls were investigated by Turkheimer et al. (2021) to study the relationship between peripheral immunity and neuroinflammation. S100B was measured as a blood–brain barrier (BBB) leakage marker, and no correlations were found between serum S100B and the kinetic parameters of BBB permeability [8].

#### 3.3.7. Neuroimaging

In the study by Lei et al. (2023), 31 BD patients, 37 MDD patients, and 61 matched HC underwent diffusion-weighted imaging (DWI). At the whole-brain level, only the MDD group showed differences compared to HC, with significantly enhanced global efficiency, local efficiency, and decreased shortest path length. MDD patients showed higher efficiency of the right amygdala, significantly elevated prefrontal-cingulate-amygdala subnetwork intensity, and higher S100B levels compared to BD patients [70].

Neuronal and glial plasma markers and brain metabolites were investigated in 10 MDD and 10 HC. Metabolite levels (N-acetyl aspartate, total choline, and total creatine) were measured in the anterior cingulate cortex (ACC) with proton magnetic resonance spectroscopy. Plasma S100B was negatively correlated with the total choline levels [72].

### 3.4. Bipolar Disorder (BD)

#### 3.4.1. Mania

Lower S100B serum levels in an exacerbation of hypomanic/manic symptoms in adolescent patients compared to healthy controls were detected, and no correlation with clinical variables, i.e., age, gender, medication status (drug-free vs. medicated), family history of psychiatric and affective disorders, symptoms severity measured using Young Mania Rating Scale, was reported [62]. Machado–Vieira et al. (2002) found opposite results: increased S100B in unmedicated patients in manic episodes and lack of a relationship with age, gender, number of previous depressive episodes, or symptoms severity (Brief Psychiatric Rating Scale; BPRS, Young Mania Rating Scale; YMRS) [93]. Andreazza et al. (2007) found higher S100B levels, both in medicated patients with manic as well as depressed episodes, compared to the euthymic group [94]. No differences in serum S100B levels in BD patients in mania compared to HC were observed, and a lack of correlation with the severity of symptoms measured using YMRS was detected. A significant decrease in S100B levels after treatment was noticed, with all patients responding well to treatment [95]. Similar results were obtained by Schroeter et al. (2002) in the study of 12 inpatients with mania, which showed a significant decrease in S100B levels after treatment. No correlation with symptoms severity was found [96].

#### 3.4.2. Depression

Comparing female inpatients with depression exacerbation in the course of bipolar disorder (*n* = 16) and major depressive disorder (*n* = 15), no differences were found in S100B levels at baseline. No differences were found concerning 8-weeks of medication, and no correlation was found either with depressive symptoms severity or history of stressful life events [77]. Combined total sleep deprivation and morning light therapy (TSD + LT) were applied to 26 inpatients in the depressive episode in the course of BD. Several growth factors were measured in plasma, regional grey matter volume was assessed, and BOLD fMRI neuronal responses to a moral valence decision task were recorded before and after treatment. S100B levels did not differ between responders and non-responders. S100B levels were not correlated with any of the studied brain morphometric data (GM volumes nor BOLD fMRI measures) [97]. The other study by Benedetti et al. (2016), using diffusion tensor imaging measures of white matter (WM) microstructure in patients with depression episodes, did not find any correlation with serum S100B and axial, radial, mean diffusivity, and fractional anisotropy white matter microstructure [98].

#### 3.4.3. Euthymia

In the large study of remitted/partially remitted monozygotic twins from Denmark’s national registries (*n* = 115 affected participants, *n* = 49 in the high-risk group, *n* = 40 in the low-risk group), no differences between groups in S100B levels were detected. Higher S100B levels were correlated with an overall poorer cognitive performance, worse working memory (recall of fewer words), and executive function (a longer time when performing TMT-B) [99].

In the study by Valiati et al. (2022), S100B protein levels were detected only in 14% of samples. This cross-sectional study compared S100B levels between MDD, BD, SCH, GAD, and HC. No differences between groups with regard to S100B levels were detected, probably caused by small groups of subjects due to the low detection rate [71].

Mesman et al. (2015), in a 12-year longitudinal study on the offspring of bipolar patients, found S100B levels in adolescent patients did not differ with HC. Still, during observation, when participants reached adulthood, S100B levels became significantly elevated. Protein level was not correlated with psychopathology state [100]. Increased levels of S100B in euthymic outpatients (*n* = 17) were also detected in the study by Haenisch et al. (2014); no analyses with any clinical variables were performed [101].

In a recent study, Knorr et al. (2022) performed a one-year longitudinal observation of remitted patients (*n* = 85) diagnosed with bipolar disorder along with HC (*n* = 44). In total, 50% of patients experienced an affective episode during a year of observation. Biological samples were withdrawn at baseline (T0), at the time of affective episode (T1), following remission (T2), and in the whole study group after a year (T3). Different Alzheimer’s disease-related biomarkers were evaluated. No differences in S100B levels between BD patients compared to healthy controls at baseline as well as T3 follow-up were noticed [102].

### 3.5. Autistic Spectrum Disorder (ASD)

Elevation in peripheral S100B levels in autistic children compared to healthy controls have been reported by several studies [103,104,105,106], while others found no differences between ASD children and healthy controls in S100B levels [107,108,109,110]. Tunca et al. (2022) compared ASD patients with healthy controls and autistic children receiving the Pervasive Developmental Disorders Supportive Training Program (ASD-NPI-individualized educational program). S100B levels in ASD-NPI children were comparable with healthy controls, while levels were elevated in ASD children without behavioral intervention [106].

Higher S100B levels in children with severe ASD than in mild-to-moderate autism were detected [103,106]. No correlation of S100B levels with severe symptoms of ASD was seen in studies using the Childhood Autism Rating Scale (CARS) [104,107,108].

A lack of correlation of S100B with age in ASD patients was reported in several studies [106,108,110].

In the study by Tomova et al. (2019), on a large group (*n* = 93) of autistic boys, a lack of correlation of S100B levels with behavioral symptoms measured with Autism Diagnostic Interview-Revised (ADI-R) and the Autism Diagnostic Observation Schedule, Second Edition (ADOS-2) scales were found. Plasma S100B concentration correlated with urine serotonin and calprotectin from stool samples [110].

Serum inflammatory markers, including S100B, were measured in a randomized, double-blinded, placebo-controlled pilot trial on probiotic and oxytocin combination therapy in ASD. Baseline S100B levels positively correlated with problem behaviors: irritability and hyperactivity/non-compliance were assessed using the Aberrant Behavior Checklist [111]. A meta-analysis recently published by Zheng et al. (2020), out of 10 analyzed studies, included three articles in Chinese (which was an exclusion criterion in our review), one report which was not found in our search in EMBASE, PubMed and Web of Science databases, and one article which studied autoantibodies against S100B using the Western Blot technique [112].

### 3.6. Attention-Deficit/Hyperactivity Disorder (ADHD)

Only five reports concern S100B levels in Attention Deficit-Hyperactivity Disorder (ADHD), and three of them are performed on the same study group: 35 children diagnosed with ADHD, including 21 medication-naïve and 14 medicated patients. The control group consisted of 21 matched healthy children [113,114,115]. No differences in S100B levels were detected between study groups. No significant correlations of S100B levels with age, BMI, IQ, allergy severity, or current socio-economic status of the father were detected. Children without oppositional or conduct problems [114], as well as hyperactivity [115], had lower S100B levels. S100B levels did not correlate with any parameters of the Continuous Performance Test (CPT) [115]. A significant positive correlation between serum S100B and maternal smoking during pregnancy among children with ADHD was detected; no correlation was found in the control group [113].

Liu et al. (2014) recruited 240 preschool children living in Guiyu, Guangdong, a city with a 30-year history of unrestricted e-waste disposal, significantly increasing exposure to heavy metals. The frequency of ADHD in the study group was higher (18,6%) than average. Boys had higher S100B levels than girls. In the group with elevated lead, S100B levels positively correlated with ADHD symptoms, hyperactivity/impulsivity, inattention, hyperactivity index, and antisocial behavior. In the low lead group, S100B negatively correlated with inattention and impulsivity-hyperactivity [116].

In the clinical trial involving 62 medication-naïve children with ADHD and 65 healthy controls, a significant elevation in baseline S100B levels in ADHD was detected. Combined therapy with methylphenidate, melatonin, and omega-3 fatty acids was administered. Three measures: baseline, after three (T3), and six (T6) months of therapy were performed. A significant increase in S100B levels between baseline and T3 and sustained elevated S100B at T6 was observed, along with an improvement in attention scores [117].

### 3.7. Suicide

Suicide is a transdiagnostic phenomenon in psychiatry. Thus, some studies are performed on S100B levels regarding suicidal behavior across different diagnoses.

Cantarelli et al. (2015) compared metabolic parameters, S100B, CRP, and BDNF levels in the group of patients with mood disorders (BD *n* = 37, MDD *n* = 13) who had a suicide attempt during the last 15 days, and patients (BD *n* = 29, MDD *n* = 7) who had no lifetime history of a suicide attempt. BD patients were in current mania or depression episodes. No differences were found in S100B levels between the two studied groups [118].

Serum S100B levels were measured in the adolescent groups with acute psychosis (*n* = 40), mood disorders (*n* = 40), and HC (*n* = 20). The Brief Psychiatric Rating Scale for Children (BPRS-C) suicidality subscale was used to evaluate suicidal ideation. S100B levels were correlated with the severity of suicidal ideation in patients, independent of psychiatric diagnosis [119]. S100B plasma levels after 6–8 weeks of treatment with antipsychotics showed a negative correlation with the number of suicide attempts in early onset schizophrenia-spectrum adolescents [43].

## 4. Discussion

The present review summarizes the findings on circulating S100B levels (serum or plasma) in five major psychiatric disorders: schizophrenia, major depressive disorder, bipolar disorder, autistic spectrum disorder, and attention-deficit/hyperactivity disorder. Our literature search retrieved 111 original articles focused exclusively on circulating S100B in these five major psychiatric disorders. The majority of the studies were performed on schizophrenia (*n* = 49) and major depressive disorder (*n* = 35), and less numerous research were conducted on bipolar disorder (*n* = 13), ASD (*n* = 10), and ADHD (*n* = 5). Three studies aimed to correlate S100B levels with suicidal behavior. This review presents a broader range of studies compared to recent meta-analyses due to less robust inclusion criteria. We also aimed to show the immense clinical variability of the analyzed groups of patients. The article encompasses studies on heterogeneous psychiatric groups at different stages of the illnesses. Clinically and biologically, there is a considerable difference between first-onset drug-naïve patients in exacerbation of symptoms and chronic patients in the remitted state or bipolar disorder patients in depression or mania. We highlighted and detailed the clinical differences between the study groups.

In the cross-sectional comparisons of patient groups with healthy controls, most of the studies consistently reported elevated S100B levels in both drug-naïve/drug-free or SCH-medicated patients [10,11,12,13,14,15,16,17,18,19,20,21,22,23,24,25,26,27,28,29,30,31,32,33,34,35], but also no differences [34,37,38], or lower S100B, in SCH compared to HC [39,40] were reported. Results of the studies in MDD are more conflicting, with fewer publications concerning first-episode patients [58,59,60,61], showing elevated [58,59,60,61,63,64,65], decreased [61,62,72,73], or no differences [60,61,66,67,68,69,70,71] in S100B levels compared to HC. In BD, studies are conducted in manic [62,93,94,95,96] or depressive episodes [77,97,98], as well as in euthymia [71,99,100,101,102], also with inconsistent results; however, a recent meta-analysis confirmed increased S100B levels in bipolar disorder [120]. In ASD, elevation [103,104,105,106], no differences [107,108,109,110] in S100B levels were reported, and parallel results were found in ADHD studies (no differences [113,114,115] or higher [116,117] S100B levels in patient groups), though in ADHD, three out of five publications were performed on the same group, analyzing different clinical aspects [113,114,115].

Longitudinal studies on treatment influence on S100B levels were also published. We can notice high heterogeneity of the studied groups (with regard to diagnosis, duration of the illness, and drug status: drug-naïve/free or medicated), as well as different pharmacological, non-pharmacological, or adjuvant interventions. In most of the reports, a decrease [12,19,45,47,48,49] or no differences [13,15,23,32,33,39,41,42,43,44,45,46,58,62,64,65,67,77] in S100B concentration were reported, and some correlations of S100B with treatment response were found [73,78,79].

Relationships of S100B with demographic and clinical parameters were studied. In the majority of the studies, a lack of correlation with age [10,13,15,30,33,35,36,40,50,60,62,64,65,69,73,74,106,108,110,114] and gender [10,13,15,22,27,30,33,40,50,52,62,73,74] was observed, both in patient groups and healthy controls. Correlations with the severity of the illness or specific groups of symptoms were reported in each disorder. Still, results are not unequivocal, and correlation analyses revealed positive or negative relationships between S100B and clinical variables. Another problem is a relatively large number of scales used to assess the severity of particular symptoms or symptom clusters in each disease.

S100B levels were also studied in the context of cognitive functions [10,17,28,45,54,115], event-related potentials [64,69,90], neuroimaging [11,52,70,72,97,98], and electroconvulsive therapy [83,85,86,87,88,89]. Finally, suicidal behavior, regardless of diagnosis status, was investigated concerning its correlation with S100B levels, also with inconsistent results, where no relationships [118], positive correlation with severity of suicidal ideations [119], or negative correlation with the number of suicidal attempts [43] were found.

S100B is widely used in neurological disorders as a biomarker of blood–brain barrier leakage in mild traumatic brain injury, ischemic stroke, and spontaneous subarachnoid hemorrhage [121,122]. Although there are numerous studies on BBB permeability in psychiatric conditions [123,124], S100B levels are only indirect measures of BBB dysfunctions, and, concerning the peripheral expression of S100B protein, its disturbances could not be directly connected to BBB dysfunctions. A recent study on the dynamics of brain barrier leakage using PET radioligands targeting found a negative association between peripheral inflammation and radiotracer perfusion into and from the brain parenchyma, and CSF. No association of this effect with circulating S100B levels was confirmed in the study [8].

## 5. Conclusions

Numerous studies of different designs were published on circulating S100B levels in five major psychiatric disorders with a shared genetic background. Meta-analyses were performed for schizophrenia [125,126,127], mood disorders [128,129,130,131,132,133], and autism spectrum disorder [111], showing elevated S100B concentrations in the studied illnesses. Only in a minority of studies were no differences or decreased S100B levels reported compared to healthy controls. Longitudinal studies on treatment effects with various pharmacological agents show no influence on S100B in the most presented studies. Different and often opposite results on correlations with symptom severity measured using numerous clinical scales are reported in all five psychiatric disorders included in the this review. Some studies report difficulties in S100B measures; also, differences in analytical techniques (standard ELISA or automated immunoassays) are present.

Alterations in peripheral S100B levels reported in psychiatric disorders seem to be non-disease- or trait-specific.

## Data Availability

Not applicable.
